# cDNA array-CGH profiling identifies genomic alterations specific to stage and *MYCN*-amplification in neuroblastoma

**DOI:** 10.1186/1471-2164-5-70

**Published:** 2004-09-20

**Authors:** Qing-Rong Chen, Sven Bilke, Jun S Wei, Craig C Whiteford, Nicola Cenacchi, Alexei L Krasnoselsky, Braden T Greer, Chang-Gue Son, Frank Westermann, Frank Berthold, Manfred Schwab, Daniel Catchpoole, Javed Khan

**Affiliations:** 1 Oncogenomics Section, Pediatric Oncology Branch, Advanced Technology Center, National Cancer Institute, 8717 Grovemont Circle, Gaithersburg, MD 20877, USA; 2 Department of Internal Medicine, College of Oriental Medicine, Daejeon University, Daejeon 301-724, Korea; 3 Department of Cytogenetics, German Cancer Research Center, Im Neuenheimer Feld 280, D-69120 Heidelberg, Germany; 4 Department of Pediatrics, Klinik für Kinderheilkunde der Universität zu Köln, Joseph Stelzmann Straße 9, D-50924 Köln, Germany; 5 Tumour Bank, The Children's Hospital at Westmead, Locked Bag 4001, Westmead, NSW, 2145, Australia

## Abstract

**Background:**

Recurrent non-random genomic alterations are the hallmarks of cancer and the characterization of these imbalances is critical to our understanding of tumorigenesis and cancer progression.

**Results:**

We performed array-comparative genomic hybridization (A-CGH) on cDNA microarrays containing 42,000 elements in neuroblastoma (NB). We found that only two chromosomes (2p and 12q) had gene amplifications and all were in the *MYCN *amplified samples. There were 6 independent non-contiguous amplicons (10.4–69.4 Mb) on chromosome 2, and the largest contiguous region was 1.7 Mb bounded by *NAG *and an EST (clone: 757451); the smallest region was 27 Kb including an EST (clone: 241343), *NCYM*, and *MYCN*. Using a probabilistic approach to identify single copy number changes, we systemically investigated the genomic alterations occurring in Stage 1 and Stage 4 NBs with and without *MYCN *amplification (stage 1-, 4-, and 4+). We have not found genomic alterations universally present in all (100%) three subgroups of NBs. However we identified both common and unique patterns of genomic imbalance in NB including gain of 7q32, 17q21, 17q23-24 and loss of 3p21 were common to all three categories. Finally we confirm that the most frequent specific changes in Stage 4+ tumors were the loss of 1p36 with gain of 2p24-25 and they had fewer genomic alterations compared to either stage 1 or 4-, indicating that for this subgroup of poor risk NB requires a smaller number of genomic changes are required to develop the malignant phenotype.

**Conclusions:**

cDNA A-CGH analysis is an efficient method for the detection and characterization of amplicons. Furthermore we were able to detect single copy number changes using our probabilistic approach and identified genomic alterations specific to stage and *MYCN *amplification.

## Background

Neuroblastoma (NB) is one of the most common pediatric solid tumors, and accounts for 7–10% of all childhood cancers. The prognosis of patients with NB varies according to the stage, age and *MYCN *amplification status. Stage 1 disease is essentially curable, whereas patients with stage 4 disease, in particular those with *MYCN *amplification, remain largely incurable despite advances in cancer therapeutics [1]. Genomic alterations in NB have been investigated by cytogenetic, and molecular methods including spectral karyotyping and metaphase comparative genomic hybridization (M-CGH) [2-6]. Based on these studies several genomic alterations have been reported to correlate with prognosis including amplification of the *MYCN *oncogene (found in 30% of NB) [1,7], gains of 17q (>50%) and loss of 1p36 (30–35%) [1,8,9]. Other recurrent changes including losses of 3p, 4p, 9p, 11q, and 14q, as well as frequent gain of chromosome 7 have also been suggested to have relevance to the development and progression of these tumors [9].

Recently array-based CGH (A-CGH) on BAC and cDNA microarrays has been used to investigate the genomic alterations with high resolution [10-14]. cDNA A-CGH has been successfully utilized to detect amplification and to investigate the direct effects of genomic changes over gene expression level by using the same microarray for both A-CGH and gene expression analysis [14-16]. In this study, we applied A-CGH, on cDNA microarrays containing 42,000 elements, to systematically identify common aberrant genomic alterations in NB of various stages. We have applied a probabilistic approach to detect single-copy losses and gains of chromosomal regions. Our study has three principal aims: 1) Detection and high resolution mapping of amplicons in NB. 2) Detection of low copy number genomic alterations using a probabilistic approach. 3) Establishing a map of genomic imbalances in NB profiling samples with good (stage 1) and poor (stage 4 with or without *MYCN *amplification) prognosis.

## Results

### Amplicon Mapping by A-CGH

**Figure 1:**
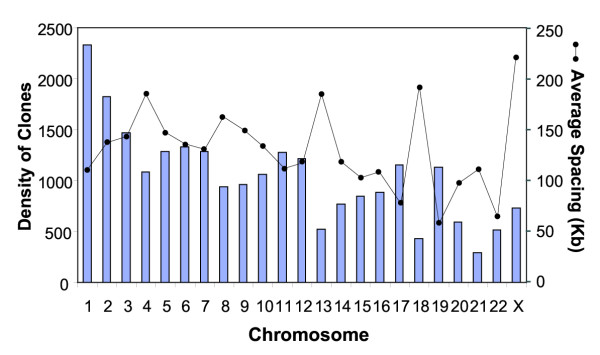
**Distribution of cDNA clones in our microarray. **Total 23975 unique UniGene clusters remained from the initial 42591 clones after quality filtering. Number of clones in each chromosome was represented in gray bar on the left side. Average spacing (chromosome size/number of clones in the chromosome) was represented in black dot on the right side.

**Figure 2:**
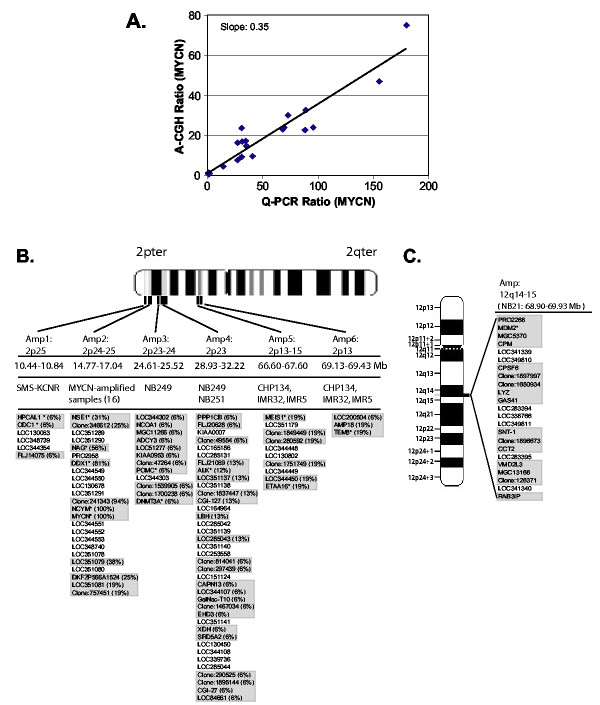
**Amplifications in *MYCN *amplified samples**. **A. **Regression analysis of *MYCN *ratio (sample *vs*. normal) obtained from A-CGH and real-time Q-PCR in all of neuroblastoma samples including 13 cell lines and 32 primary tumors. The slope of the regression line is 0.35 indicating that an observed A-CGH ratio of 2 is equivalent to a Q-PCR ratio of 5.7. **B. **Independent amplicons in chromosome 2p. All amplified genes are listed under each amplicon in genome order. Map position, genome sequence position (Mb) and samples containing the specific amplicon are listed for each amplicon. The percentage of the *MYCN *amplified samples harboring these amplicons are shown in brackets following the gene name for all clones present in our microarray (gray), the remainder of the clones are predicted genes found in the NCBI database  that are mapped between the boundaries of the amplicon. Amplicons were selected based on the criteria of A-CGH ratio ≥2 for at least two contiguous clones in genome sequence order. In cases where a single clone has a ratio <2 but the ratio of its adjacent clones is greater than 2, that single clone was still considered as a part of amplicon. *: previously reported amplification. **C. **Amplicon in chromosome 12q in tumor NB21.

Totally around 24,000 qualified array cDNA clones were applied for data analysis in 12 NB cell lines and 32 NB primary tumor samples (see Table 1 for sample information). Fig. 1 shows the number of clones as well as the average spacing for each chromosome. We first determined the sensitivity of A-CGH to detect the copy number of highly amplified genes. We here chose *MYCN *since it is the most commonly amplified gene in NB and correlates with the biological behavior of these tumors. Fig. 2A shows the linear regression plot of the *MYCN *amplification results from A-CGH and Quantitative-PCR (Q-PCR). We found that the slope of the fitting line was 0.35, and therefore an observed ratio of 2 by A-CGH corresponds to Q-PCR ratio of ~6. In order to identify the amplified regions, we initially selected genes with A-CGH ratio ≥2 for at least two contiguous clones in genome sequence order. Only two chromosomes (2p and 12q) showed amplifications by this criterion exclusively in the *MYCN *amplified samples. Focusing on 2p (Fig. 2B), we found 6 independent non-contiguous amplicons (10.4–69.4 Mb). For the *MYCN *amplicon, the largest contiguous region was 1.7 Mb and bounded by *NAG *and an EST (clone: 757451) in three tumor samples, whilst the smallest region was 27 Kb including an EST (clone: 241343), *NCYM*, and *MYCN*. We identified 9 previously reported co-amplified genes (*HPCAL1*, *ODC1*, *NSE1*, *NAG*, *DDX1*, *NCYM*, *POMC*, *DNMT3A*, *ALK, MEIS1, TEM8*) [16,20-27], and detected the novel amplification of several known genes (*NCOA1*, *ADCY3*, *PPP1CB*, *CGI-127*, *LBH*, *CAPN13*, *GalNac-T10*, *EHD3*, *XDH*, *SRD5A2*, *CGI-27*, *AMP18*) and ESTs. Three of the cell lines (CHP134, IMR-5 and IMR-32) contained two amplicons in 2p13-15. The first (66.6–67.6 Mb) included previously reported amplified gene *MEIS1*, and the size of the second amplicon was 0.3 Mb (69.1–69.4 Mb), which was bounded by *LOC200504 *and *TEM8*. In addition to chromosome 2p, we identified another amplicon on 12q14-q15 in a single tumor (NB21); bounded by *PRO2268 *(68.9 Mb) and *RAB3IP *(69.9 Mb) containing one previously reported amplified gene (*MDM2*) [28] as well as several novel amplifications (*CPM*, *CPSF6*, *LYZ*, *GAS41*, *SNT-1*, *CCT2*, *VMD2L3*, *and RAB3IP*) (Fig. 2C). We verified the amplification of *NSE1*, *NAG*, *DDX1*, *MYCN *and *TEM8 *by Q-PCR (data not shown). Simultaneous gene expression profiling by using the same cDNA arrays for all samples showed that 47% of the amplified genes correlate with gene expression (using a correlation coefficient cutoff 0.5; data not shown).

**Table 1:** Summary of Neuroblastoma Information

Sample No	Sample Label	Age (yr.mo)	Sex	Diagnosis	MYCN	Source
C1	CHP-134B	1.1	M	4	+	NCI
C2	GI-LI-N	1.11	M	4	+	ICLC
C3	IMR-32	1.1	M	4	+	ATCC
C4	IMR-5	ND	ND	ND	+	ICLC
C5	LAN-1	2	M	4	+	ICLC
C6	LAN-5	0.4	M	ND	+	NCI
C7	SK-N-BE(2)	2.2	M	4	+	ATCC
C8	SK-N-DZ	2	F	ND	+	ATCC
C9	SMS-KCNR	1.2	M	4	+	NCI
C10	SH-SY5Y	4	F	4	-	ATCC
C11	SK-N-AS	8	F	4	-	ATCC
C12	SK-N-FI	11	M	ND	-	ATCC
C13	SK-N-SH	4	F	4	-	ATCC
T1	NB19	12.9	F	1	-	DZNSG
T2	NB20	1.3	M	1	-	DZNSG
T3	NB229	0.3	M	1	-	CHTN
T4	NB248	0.6	M	1	-	CHW
T5	NB29	0.3	M	1	-	DZNSG
T6	NB33	1.5	F	1	-	DZNSG
T7	NB34	1.2	M	1	-	DZNSG
T8	NB43	1.1	F	1	-	DZNSG
T9	NB44	1.6	M	1	-	DZNSG
T10	NB5	0.3	M	1	-	DZNSG
T11	NB7	1.3	F	1	-	DZNSG
T12	NB9	1.1	M	1	-	DZNSG
T13	NB16	3.11	F	4	-	DZNSG
T14	NB205	3.11	F	4	-	CHTN
T15	NB217	2	M	4	-	CHTN
T16	NB24	0.7	M	4	-	DZNSG
T17	NB246	3.7	M	4	-	CHW
T18	NB247	1.5	M	4	-	CHW
T19	NB26	1	M	4	-	DZNSG
T20	NB30	0.11	F	4	-	DZNSG
T21	NB31	1.4	F	4	-	DZNSG
T22	NB32	1.2	M	4	-	DZNSG
T23	NB35	2.7	F	4	-	DZNSG
T24	NB8	4.7	M	4	-	DZNSG
T25	NB14	0.11	M	4	+	DZNSG
T26	NB21	5.3	M	4	+	DZNSG
T27	NB249	0.8	M	4	+	CHW
T28	NB251	0.9	F	4	+	CHW
T29	NB252	0.10	F	4	+	CHW
T30	NB266	2	F	4	+	CHW
T31	NB27	10.6	M	4	+	DZNSG
T32	NB28	1.8	F	4	+	DZNSG

Abbreviations used are ND: not determined ATCC: American Type Culture Collection, CHTN: Cooperative Human Tissue Network CHW: The Children's Hospital at Westmead, Australia, DZNSG: German Cancer Research Center, Heidelberg, ICLC: Interlab Cell Line Collection, NCI: National Cancer Institute, NIH. ^1^Naming convention "C" denotes cell lines, "T" tumors, such that C1 is cell line number 1.

### Detection of low-level DNA copy number alterations

**Figure 3:**
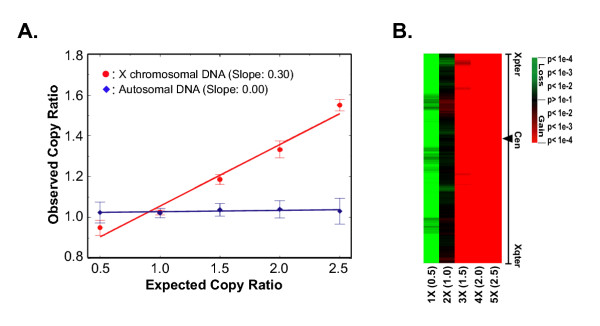
**Sensitivity of A-CGH to detect the low-level copy number alteration**. **A. **Measurement of X-chromosomal copy number. A-CGH was performed to analyze the copy number of genes in the X-chromosome. Female DNA (XX) was used as the reference DNA. Male DNA (XY), female DNA (XX), and DNA samples containing different number of X-chromosome (XXX, XXXX, XXXXX) were used as test DNA, with an expected ratio of test/reference of 0.5, 1.0, 1.5, 2.0, and 2.5 respectively for X-chromosome. Mean fluorescence ratios (±SEM) of autosomal DNAs (blue diamonds) and X-chromosomal DNAs (red circles) from each experiment are shown. The slope of the regression line is 0.3. **B. **Visualization of p-values derived from the topological statistics as described in the Methods along the X-chromosome from samples containing different X-chromosomal copy numbers. Each column represents a different experiment; and each row represents the p value for the alteration at a given SW-locus (a sliding window of 40 adjacent clones, details in Methods), ordered by genome map position from Xpter to Xqter. Red represents gain and green loss. The intensity of the color shows the level of significance according to the p-value shown in the color scale.

To test the sensitivity of A-CGH to detect single copy number changes, we performed A-CGH with DNA from cell lines containing different numbers of X chromosomes (1–5 copies) [12] and compared them to a sample with 2 copies of X chromosomes. The observed mean fluorescence ratio of all clones across X chromosome was calculated (Fig. 3A). For single copy deletions we observed an A-CGH ratio 0.9 (expected 0.5). The regression slope was 0.3, similar to that for the *MYCN *above (Fig 2A). The underestimation of the expected ratio by A-CGH demonstrated that it is difficult to detect single-copy changes using pre-set threshold-based approaches.

In order to increase the sensitivity for detecting low copy number changes, we applied a probabilistic approach utilizing t-statistics and the local genomic sequence mapping information of each of the cDNA clones on our arrays. To validate our method, we re-analyzed the A-CGH data generated from the cell lines containing 1–5 copies of the X chromosome as described above, and we were able to detect a single copy loss and gain of X chromosome where the expected ratio was 0.5 and 1.5 respectively (Fig. 3B). In addition, we used the reported results from the literature as an independent validation. The cell line SK-N-AS is deleted within 1p36.2-p36.3, which has been investigated by FISH and southern blot analysis [29,30]. The proximal SK-N-AS deletion breakpoint was mapped to between *NPPA *and *PLOD*, while the distal breakpoint is proximal of *CDC2L1*. The deletion detected by our method is bordered by *KIAA0495 *and *CTNNBIP1*, which is within the region reported. They are in a very good accordance. In addition, we also compared the 17q gain results for four NB cell lines (CHP134, IMR-5, SMS-KCNR, and SK-N-AS) with the results in literature by FISH and Q-PCR [31]. Our results confirmed the gains in 17q for all 4 cell lines and the loss in 17p in SK-N-AS detected by FISH.

**Figure 4:**
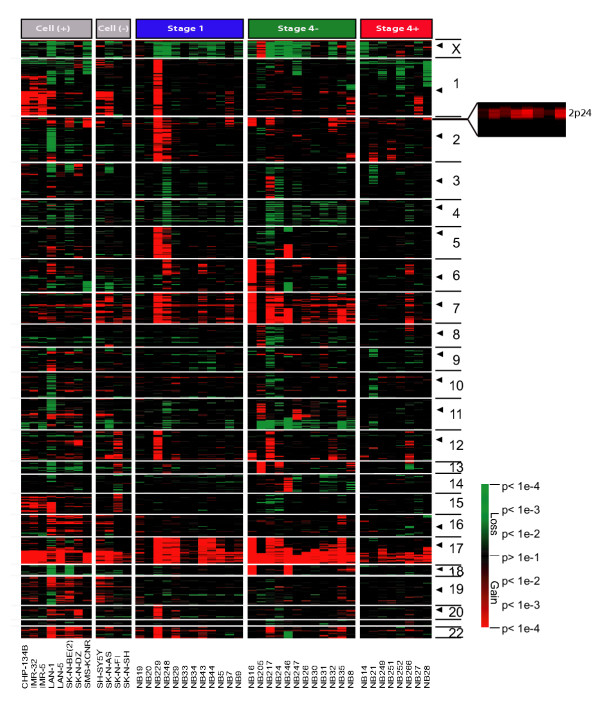
**Genome-wide analysis of DNA copy number alteration by A-CGH. **Samples were grouped based on sample type, *MYCN *amplification status and tumor stage. Each column represents a different sample; and each row represents a p-value of a given SW-locus using a sliding window of 40 adjacent clones, ordered in genome order across the whole genome. Black triangles on the right side of image represent centromere positions. Cell-: cell line without *MYCN *amplification; Cell+: cell line with *MYCN *amplification; Stage 4-: tumor in stage 4 without *MYCN *amplification; Stage 4+: tumor in stage 4 with *MYCN *amplification. On the right is shown an enlarged view of the region around the *MYCN *gene (2p24) for the amplified NB samples.

We next analyzed the A-CGH data using this method to detect genome-wide alterations of DNA copy number in our NB samples. Using this t-statistics, we identified DNA copy number alterations that involved the majority of the chromosomes in both primary tumors and cell lines (Fig. 4). We confirmed previously reported genomic changes, including gains of whole chromosome 1, 2, 6, 7, 8, 12, 13, 17, 18 and 22, and losses of 3, 4, 9, 11, and 14; partial gains of 1q, 2p, 11p, 12q and 17q; partial losses of 1p, 3p, 4p, 9p, 11q and 14q [9,32]. The most common changes were losses on chromosome 1p, 4 and 11q; gains on 2p, 7, and 17q.

### Stage specific genomic alterations

**Figure 5:**
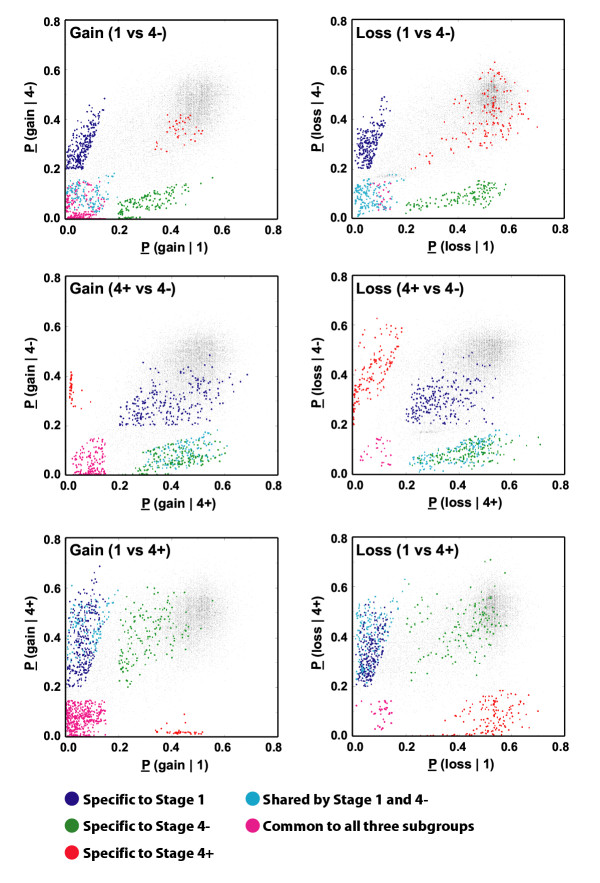
**Genomic alterations specific to stage and *MYCN *status. **Shown is the graphic depiction of the average p-values (P) of genomic alteration of each SW-locus (using a sliding window of 20 adjacent clones) within each tumor subgroup. All possible pair-wise comparisons among the three subgroups for gains or losses (stage 1 vs 4-, 4+ vs 4- and 1 vs. 4+) are shown. The frequency of alteration is estimated by P such that P = 0.15 is equivalent to a frequency of 70% and the lower the P the higher the frequency (details in Methods). Different colors were used to represent different clusters. Magenta: loci common to all groups; cyan: common to 1 and 4-; blue: 1 specific; green: 4- specific; red: 4+ specific, and black: all remaining loci. Colored dots were enlarged for easier visualization.

In order to identify the recurrent regions of genomic alterations that are specific to stage and *MYCN *amplification status, we partitioned the tumors into three subgroups (stage 1, stage 4 *MYCN *not amplified (4-) and amplified (4+)), and analyzed the frequency of genomic changes at each SW-locus (as defined in Methods) for each subgroup. Since cell lines may contain tissue culture related genomic alterations, we only used primary NB tumor samples for this analysis. The frequency of alterations for a given SW-locus was estimated using the average probability (P) value as described in the Methods. Fig. 5 shows the graphic depiction of the P associated with each SW-locus for all possible pair-wise comparisons among the three subgroups for gains or losses: Stage 1 *vs*. 4-, 4+ *vs*. 4- and 1 *vs*. 4+. As expected, since the majority of the loci show no change, they were plotted to values of co-ordinates around (0.5, 0.5) (shown in black). Regions altered in both classes with similar frequency are plotted close to the y = x diagonal, whilst off-diagonal points represented regions primarily altered in one or the other of the classes (termed differential imbalance). Loci that plot around (0,0) reflect loci altered in both groups (termed common regions). The colored points were selected by using our criterion for "common" and "specific" SW-locus (see Methods). In summary, we found alterations that were common to all three subgroups, which included gain of 7q32, 17q21, 17q23-24 and loss of 3p21. We also found genomic imbalances that were specific for each of the subgroups and common regions of gain for stage 1 and 4- tumors. Of note there were no shared alterations of 4+ with 1 or 4- besides the regions common to all. A detailed description and map positions for all these recurrent regions are provided in the Table 2, and a graphic representation of these imbalances is shown in Fig. 5, where we will discuss in detail in the discussion section.

**Table 2:** Recurrent regions related to MYCN status and stage

	**Imbalance**	**Chr.**	**Cytoband**	**Start (Mb)**	**End (Mb)**	**Clone No.**
**Common to 1, 4-, & 4+**	gain	7	7q32	133.19	137.05	26
	gain	17	17q21	42.13	53.53	157
	gain	17	17q23	55.41	61.8	99
	gain	17	17q24	65.59	73.44	70
	loss	3	3p21	45.72	46.77	13
**Shared by 1 & 4-**	gain	7	7p14	32.41	32.83	11
	gain	7	7q11	69.89	72.34	28
	gain	7	7q36	148.74	152.97	39
	gain	17	17q12	35.45	36.41	15
	gain	17	17q21	39.56	40.03	16
**Specific to 1**	gain	2	2p22	32.23	33.33	12
	gain	17	17p13	2.12	2.59	11
	gain	17	17p13	5.67	7.17	22
	gain	17	17p13	8.23	9.42	17
	loss	8	8p12	38.32	39.84	12
	loss	8	8q22-23	109.54	116.64	13
	loss	11	11q12	60.87	63.09	29
	loss	14	14q12	28.94	31.32	11
	loss	14	14q23	58.04	58.76	12
	loss	19	19q13	43.4	44.05	13
**Specific to 4-**	gain	7	7p15	24.26	25.89	12
	gain	7	7q34	139.53	141.16	22
	loss	11	11q21	96.25	96.83	11
	loss	11	11q22	108.12	110.64	11
	loss	11	11q23	114.31	120.39	51
	loss	11	11q24	125.28	126.76	20
	loss	11	11q25	131.98	134.08	19
**Specific to 4+**	gain	2	2p24-25	10.97	20.08	38
	loss	1	1p36	6.16	13.42	78
	loss	1	1p36	15.22	16.13	20

## Discussion

### Amplicons in Neuroblastoma

In our study we found that unlike breast cancers [14] NBs do not have a wide variety of different amplicons or amplified genes. We identified 6 independent amplicons on 2p and one on 12q and precisely defined boundaries for all amplicons. Several genes related to angiogenesis and oncogenesis were in these novel amplified regions including *TEM8 *(tumor-specific endothelial marker), mapped to 2p13.1, which has been shown to have elevated expression during tumor angiogenesis [33]. Indeed this gene was recently reported to be amplified in the cell line IMR-32 in accord with our data [27]. The gene is expressed in human endothelium and has been implicated in colorectal cancer. Our present study showed *TEM8 *was amplified and over-expressed (data not shown) in several neuroblastoma cell lines. The significance of amplification of *TEM8 *in neuroblastoma cell lines but not endothelial cells raises an intriguing possibility that these tumor cells themselves contribute to the angiogenic process and requires further investigation. We also identified amplification of *GAS41 *(glioma amplified sequence) mapped to 12q14-q15 in one tumor sample. *GAS41*, a transcription factor ubiquitously expressed with the highest expression in human brain, was previously shown frequently amplified in human gliomas [34]. *ALK *(anaplastic lymphoma kinase) receptor, an oncogene and reported highly expressed in neuroblastoma [26], was identified to be amplified in two of our tumor samples. In addition to these genes, most of those newly identified amplified genes have not been implicated previously in neuroblastoma tumorigenesis and progression; therefore a further characterization of these genes might provide the biological insights to neuroblastoma biology. Interestingly, all amplicons occurred in *MYCN *amplified samples, and we have not found a single amplicon in *MYCN *single copy samples.

Additionally, using our search criterion (A-CGH ratio >2 corresponding to copy number >6 see above) we found no evidence of amplifications in other chromosomal regions. This was in conflict with a study by Satito-Ohara et al. who found evidence of high level gains 9 NB cell lines and amplification as evidenced by a homogeneous staining region (HSR) in one line [35]. This difference could be explained by potential artifacts that arise in cell lines in tissue culture or be as a result of under detection by our study because of the relatively small number of tumor samples in our study, and would require confirmation in a larger sample set.

### Detection of low level of genomic changes

In this study, we have applied a t-statistics-based method to explore genomic alterations in cancer from data generated by A-CGH on a cDNA microarray platform. Our method efficiently dealt with the low sensitivity of cDNA microarrays to detect low copy changes. The microarrays we utilized contain 42,000 clones, containing around 24,000 unique UniGene clusters with an average coverage of one cluster every 125 Kb. Underestimation of DNA copy number ratios by cDNA A-CGH data made it difficult to detect low level of gains and losses using ratio threshold based approaches, which was addressed in our study and previous reports [14-16]. The algorithm we have implemented included an efficient noise reduction strategy by combining ratios within a sliding window of clones as has been previously described [13,14]. However the incorporation of t-statistics demonstrated several advantages over the sole reliance of moving average for detecting genomic changes. It provides confidence levels for detecting genomic changes at each DNA location (SW-locus) in terms of p-values. This has theoretical advantages over the original raw ratio, because it incorporates an estimate of possible statistical errors in the analysis by giving a p-value attached to each genomic change within a SW-locus. By this method we were able to detect 1.5 fold changes of gene copy number as shown in our X-chromosome validation experiment.

However, all these advantages are traded for a loss of resolution: genomic imbalances much smaller than the window-size cannot be detected and the boundaries of instable regions are blurred. Therefore we should choose the smallest window that has the desired level of statistical significance. The effective resolution can be obtained by analyzing the correlation of overlapping sliding windows. The integrated autocorrelation time is an estimator of the minimal distance for windows to be effectively uncorrelated [36] even when they overlap. For the sliding window t-test in our algorithm this distance can be calculated to be half the window size w, thus the number (N) of total unique Unigene clusters is reduced to 2N/w for the effectively independent measurements of the DNA copy number. Our results indicate that a window size 20 is needed in order to detect the lowest possible DNA change (one copy change) with reasonable statistical significance. According to the discussion above, this window size reduces the approximately 24,000 quality filtered unique Unigene clusters to 2 * 24,000/20 = 2400 independent estimates. This resolution is comparable to typical BAC arrays. For the stronger signals, less noise reduction is required. To detect 2-copy number DNA changes, only a small window size 5 is needed, therefore the resolution will be 4 fold higher. Although cDNA A-CGH is known not as sensitive as BAC A-CGH for the detection of low level of DNA copy number changes, currently we are able to obtain the comparable detection by using the probabilistic approach. In addition, with cDNA array it is possible to identify genomic amplification at the gene level and investigate the direct effect of gene copy number change over gene expression level in parallel, which will be addressed in future studies.

## Conclusions

In this study we explored the genomic alterations in NB from the data generated by A-CGH on a cDNA microarray platform. We have not found genomic alterations universally present in *all *(100%) three subgroups of NBs, although such a region would be interesting since it may harbor specific genes that are uniquely responsible for NB tumorigenesis. We therefore focused on commonly altered regions where >70% of tumors showed changes in a given region, for our three different subgroups (Fig. 5 and Table 2). We found only a few of imbalances occurring in all three subgroups, of which gain of 17q21-24 and loss of 3p21 have been previously described in NB biology [8,37]. Apart from these regions stage 4+ tumors did not have any other regions that commonly change with the other two stages, whereas stages 1 and 4- had several common alterations. Stage 4- tumors demonstrated several unique changes of which losses in 11q has been previously described in *MYCN *single copy NB [38] and acts as a possible marker of unfavorable phenotype independent of *MYCN *amplification [39]. Remarkably stage 4+ disease appears to have very few genomic alterations when compared with Stage 1 and 4- implying that *MYCN *amplification is sufficient to drive these tumors to an aggressive phenotype, and although other genomic changes occur, including loss of 1p36 as shown by us and others [40], it does not require extensive changes. This is in agreement with the murine *MYCN *transgenic model of NB where the *MYCN *transgene itself is enough for tumor development, but these tumors develop additional genomic changes characteristic of NB [41].

Based on these results we found that cDNA A-CGH analysis is an efficient method for the detection and characterization of amplicons. We confirmed the previously reported amplified genes and also identified novel amplifications in neuroblastoma. Furthermore our probabilistic approach allows the detection of single copy number changes from cDNA A-CGH and can be applied to other CGH platforms including BAC or oligonucleotides based arrays.

## Methods

### Tumors, cell lines, and genomic DNA

Thirty-two snap frozen neuroblastoma specimens were obtained from 12 patients with stage 1, and 20 patients with stage 4 of which 12 were *MYCN*-amplified and 8 were *MYCN *single-copy tumors. The original histological diagnoses were made at tertiary hospitals with extensive experience in diagnosis and management of neuroblastoma. Additionally, 12 neuroblastoma cell lines including 8 *MYCN*-amplified and 4 *MYCN *single copy samples were used in the study. Details of individual sample are summarized in Table 1. The conditions for cell cultures were done as described previously [17]. High molecular weight genomic DNA was extracted from interphase of a Trizol preparation for RNA extraction according to the manufacturer's instructions (Invitrogen, Gaithersburg, MD). Genomic DNA was treated with RNase A and protease (Qiagen, Valencia, CA), and purified by phenol/chloroform extraction followed by ethanol precipitation. We obtained normal genomic DNA samples (male, female or 1:1 mixture of male and female) from Promega, and genomic DNA samples containing the different numbers of X chromosomes (XXX, XXXX, and XXXXX) from the NIGMS .

### Microarray experiments

Preparation of glass cDNA microarrays was performed according to a previously published protocol [18]. Image analysis was performed using DeArray software [19]. The cDNA library containing 42,000 clones was obtained from Research Genetics (Huntsville, AL) and clones were printed on two microscope glass slides as a set. Approximately 50% of the cDNAs on the microarrays were either known genes or similar to known genes in other organisms, whereas the remainders were anonymous ESTs. For A-CGH experiments on cDNA microarrays, 20 μg of genomic DNA from neuroblastoma tumor or cell line samples were sonicated and purified with QIAquick PCR purification column (Qiagen, Valencia, CA). Three micrograms of sonicated DNA were labeled with aminoallyl-dUTP (Sigma) in a 25-μl reaction, including random hexamer (0.24 μg/μl, Roche), dATP, dCTP and dGTP (125 μM each), dTTP (25 μM), aminoallyl-dUTP (100 μM) and high concentration of Klenow fragment (2.5 U/μl, NEB). The labeling reaction was purified with QIAquick PCR purification column. Cy3 and Cy5 dyes were coupled to the reference DNA (1:1 mixture of normal male and female DNA) and sample DNA respectively. Cy3- and Cy5-labeled probes were then combined along with human Cot-1 DNA (50 μg, Invitrogen) and yeast tRNA (100 μg, Invitrogen). The mixture was concentrated and re-suspended in 32 μl of hybridization buffer (50% formamide, 10% dextran sulfate, 4 × SSC, and 2% SDS). The hybridization mix was first heated at 75°C for 10 min, then at 37°C for an hour, and finally loaded to the pre-hybridized array. The hybridization was performed at 37°C overnight. The washing procedure was performed as described previously [17].

### Real time quantitative PCR

Real time PCR was carried out using SYBR Green PCR core reagents according to the manufacturer's instructions (Applied Biosystems, Foster City, CA). Each DNA sample was analyzed in triplicate using the ABI PRISM 7000 Sequence Detector. For quantitative PCR, 10 ng of genomic DNA was used for SYBR green PCR assay. Serial dilutions of neuroblastoma cell line CHP134 DNA were used as templates for a standard curve, and the normal genomic DNA was used as a calibrator. The normalization was performed as described using *BCMA *and *SDC4 *as reference genes [20].

### Data analysis

Fluorescence ratios were normalized for each microarray by setting the average log ratio for each subarray elements equal to zero (commonly referred to as "pin-normalization"). The data was quality-filtered by removing those clones with quality lower than 0.5 in more than 20% of all the samples [19]. For the clones that passed this filter, if the quality for a specific sample is lower than 0.5, then its fluorescence ratio is replaced by the average ratio value of all other samples with the good quality. The clones were finally assigned to UniGene Cluster (Build 154 September 2002). For the UniGene clusters represented by multiple clones, mean fluorescence ratios of those clones are used. After these processes we had 23975 unique UniGene clusters remaining from the initial 42591 clones. Map positions for the cluster were assigned by Blat searches against the "Golden Path" genome assembly (; June, 2002 Freeze). Throughout this publication, all genomic coordinates are given with the respect to this assembly. Finally the clusters were sorted according to their starting position of sequence on each individual chromosome.

### Detection of single copy changes

To identify the alterations of copy number along the genome, we compared the distribution of the ratios in a sliding window of 20 clones in genomic order with a "null distribution" using a t-test. The p-value for genomic change (P_gc_) obtained in this way is assigned to the center of the sliding window (referred to as the "SW-locus" throughout this manuscript). The t-test is valid in this instance because the observed distribution of P_gc _for the loci in random order matches the expected theoretical distribution. The null-distribution used in t-test represents the unaltered part of genome. To identify the cDNA clones for the null-distribution, we start with the whole genome. The SW-loci corresponding to portions of the DNA that are amplified or deleted with a p-value smaller than 0.05 are removed recursively from the null dataset, until the null dataset is stable and there is no more amplified or deleted SW-locus in the null data set. Finally, the confidence of identified genomic alterations is visualized in a pseudo-color map in which color intensity represents the log of p-values (red for gain and green for loss).

### Estimation of frequency of genomic changes among the samples

The probabilistic approach above provides P-values for the presence of genomic alteration in a given sample. In order to estimate the frequency of a genomic alteration we can set a threshold ratio and identify how many samples have a ratio value outside this threshold in the same genomic region. The disadvantage of this method is that different ratio thresholds will give different frequencies. We therefore applied another approach to avoid the use of ratio thresholds. To determine the frequency of loss or gain that correlate with the stage or *MYCN *amplification status, we first calculated the mean of the P_gc _or P, for each group. This value is proportional to the frequency f_with change _= N_with change_/N_total _of their occurrence, where N_with change _is the number of samples in a subgroup with a given genomic imbalance and N_total _is the overall number of samples in that subgroup. This is valid as follows. For all loci in which there are no genomic imbalances the observed P_gc _will follow the flat theoretical distribution with a mean (expectation value) <P> = 0.5. Therefore, for those cases where we are sure of a genomic imbalance P_gc _is close to 0 (for example p < 0.01), whereas for the samples in which there are no changes P_gc _= 0.5. According to the formula: P = ((N_no change _× 0.5) + (N_with change _× 0))/N_total _= (1 - f_with change_) × 0.5, the lower the P the higher the frequency of a genomic change in that SW-locus. Thus the P can be used to determine the frequency of a given change e.g. P = 0.15 corresponds to a frequency of ~70% of the samples with that given change.

### Determination of recurrent regions

We first define a SW-locus with P < 0.15 in a specific subgroup as altered. This threshold corresponds to roughly a fraction of >70% of all tumor samples harboring that alteration in each subgroup. We define an altered SW-locus as common in all tumors, if the SW-locus passes the threshold for each of the three subgroups. A SW-locus is called differential in one subgroup with respect to another subgroup, if the frequency of genomic change is at least 3 times higher in one subgroup as compared to another. A SW-locus is defined as specific if the locus in one subgroup is differential with respect to each of the other subgroups; a SW-locus is defined as shared in two groups, if in both groups it is differential with respect to the third subgroup.

## Abbreviations

NB: neuroblastoma

A-CGH: array-comparative genomic hybridization

M-CGH: metaphase-comparative genomic hybridization

Stage 1-: stage 1 without *MYCN *amplification

Stage 4-: stage 4 without *MYCN *amplification

Stage 4+: stage 4 with *MYCN *amplification

BAC: Bacterial artificial chromosome

## Author's contributions

QC carried out all experiments and participated in data analysis. SB performed array CGH data analysis and statistical analysis. QC and SB drafted the manuscript. JSW, CCW, NC and CS were involved in the microarray production and the manuscript edition. ALK and BG were involved in the data analysis and the manuscript edition. FW, FB, MS and DC provided the tumor samples and patient information and were also involved in the manuscript edition. JK principal investigator of the project, participated in its design and is the final editor of the manuscript. All authors read and approved the final manuscript.
